# Determinants of hepatitis B virus infection among pregnant women in Bench Sheko zone, Southwest Ethiopia: a case-control study

**DOI:** 10.3389/fgwh.2024.1453231

**Published:** 2024-10-14

**Authors:** Tewodros Yosef, Ephrem Eyasu, Nigusie Shifera, Gossa Fetene Abebe, Desalegn Girma, Aklilu Habte, Ahmed Fentaw Ahmed, Adane Asefa

**Affiliations:** ^1^ School of Public Health, College of Medicine and Health Sciences, Mizan-Tepi University, Mizan-Teferi, Ethiopia; ^2^ Faculty of Health, School of Medicine, Deakin University, Waurn Ponds, VIC, Australia; ^3^ Department of Midwifery, College of Medicine and Health Sciences, Mizan-Tepi University, Mizan-Teferi, Ethiopia; ^4^ School of Public Health, College of Medicine and Health Sciences, Wachemo University, Hosanna, Ethiopia; ^5^ Department of Public Health, College of Medicine and Health Sciences, Injibara University, Injibara, Ethiopia

**Keywords:** hepatitis B virus, viral infection, pregnant women, sero-prevalence, Southwest Ethiopia

## Abstract

**Background:**

Hepatitis B Virus (HBV) infection during pregnancy poses serious risks by raising the likelihood of chronic infection in newborns by 90% and the risk of cirrhosis and liver cancer by 25% in chronic infections. This study aimed to identify determinants of HBV infection among pregnant women in the Bench Sheko zone, Southwest Ethiopia.

**Methods:**

An unmatched case-control study was conducted from May 15 to July 15, 2022, in selected health facilities of the Bench Sheko zone, Southwest Ethiopia. Medical charts were reviewed to collect the HBsAg status of participants, as all pregnant women attending antenatal care underwent routine screening. It involved 228 pregnant women (76 HBV-positive cases and 152 HBV-negative controls). Data were collected using structured questionnaires, and analyzed using SPSS 21. A multivariable logistic regression was performed to identify significant determinants of HBV infection, and statistical significance was declared at *p*-value <0.05.

**Results:**

After controlling potential confounders, having no formal education (AOR = 4.94, 95% CI: 2.01, 8.29; *P = 0.007*), urban residency (AOR = 2.56, 95% CI: 1.43, 6.86; *P = 0.010*), history of unsafe abortion (AOR = 3.87, 95% CI: 2.17, 6.98; *P < 0.001*), sharing sharp materials (AOR = 8.43, 95% CI: 5.54, 10.9; *P < 0.001*), contact with HBV-infected persons in the family (AOR = 2.18, 95% CI: 1.72, 4.87; *P < 0.001*), tribal scarification (AOR = 3.23, 95% CI: 1.24, 8.91; *P = 0.017*), and history of unsafe tooth extraction (AOR = 4.52, 95% CI: 2.18, 9.76; *P = 0.039*) were identified as significant predictors of HBV infection.

**Conclusion:**

The study identifies multiple factors contributing to HBV infection in pregnant women. Therefore, it is crucial to promote safe abortion practices and the responsible use of sharp materials, avoid high-risk contact with infected individuals within the family, raise awareness about the risks associated with tribal scarification while advocating for safer practices, and offer education on safe tooth extraction methods to reduce the risk of HBV.

## Introduction

Hepatitis B infection, caused by the Hepatitis B Virus (HBV), infects the liver and can lead to cirrhosis and hepatocellular carcinoma (HCC) (1). The incubation period is 45 days to 6 months. Around 10% of children and 30%–50% of adults with acute infection experience symptoms (2, 3). HBV, primarily transmitted through blood, semen, vaginal secretions, and contaminated fluids, poses significant health risks. Major transmission routes include sexual contact, household contact, injecting drug use, and medical exposure (1–3). Possible modes of transmission are unscreened blood transfusions, needle sharing, hemodialysis, acupuncture, tattooing, and injuries from contaminated sharp instruments (1, 2). Chronic HBV infections can lead to severe complications in 20%–30% of cases, resulting in approximately 650,000 annual deaths (2).

Globally, hepatitis B infection remains a significant health concern, with approximately 1.5 million new infections each year and 820,000 related deaths reported in 2019. The global prevalence stands at 3.8% (4). In Africa, there were 990,000 new infections and 80,000 deaths in 2019, with carrier prevalence in sub-Saharan Africa ranging from 10% to 20% (4). Ethiopia, designated a high-burden country, exhibits an HBsAg prevalence of 35.8% among chronic liver disease patients (5). Recent data from 2018 indicates a pooled prevalence of 4.7% among pregnant women, with higher rates observed in specific regions such as Gambella (7.9%) and Bench Sheko Zone (5.9%) (6).

In endemic areas, the high prevalence of HBeAg in women of reproductive age correlates with increased prenatal HBV transmission (7). HBV infection during pregnancy poses significant risks to both mother and child (8), with approximately 10%–20% of HBsAg-positive pregnant women transmitting the virus to their babies. Transmission rates approach 100% for women positive for both HBsAg and HBeAg (7), leading to newborns facing an 85%–90% risk of developing chronic liver diseases (9). Tragically, about 25% of those infected as infants or young children succumb to cirrhosis and liver cancer, often without symptoms until late-stage disease (10).

Studies in Sub-Saharan countries investigating determinants of hepatitis B virus (HBV) among pregnant women have yielded inconsistent findings. Research in Uganda found no significant relationship between HBsAg positivity and factors like scarification, number of sexual partners, blood transfusion, polygamy, surgical procedures, or marital status (11). However, other studies highlighted scarification, blood transfusion, concurrent HIV infection, multiple sexual partners, and tribal scar as significant factors associated with HBV infection (12–15).

The high prevalence of HBV among pregnant women in Ethiopia represents a significant public health concern (16). Despite government efforts to screen for viral hepatitis in pregnant women, there's limited focus on identifying the root causes of HBV infection within the health system (6, 17). Given the potential transmission from mother to child, understanding the main factors contributing to its spread among pregnant women is crucial. Thus, this study aims to identify significant determinants of HBV infection in pregnant women.

## Methods

### Study design, area, and period

A health institution-based case-control study was conducted in Bench Sheko Zone, southwest Ethiopia from May 15 to July 15, 2022. The zone is located 585 km from Addis Ababa. The zone's population was 653,986, with 330,459 females and 323,527 males. Among them, 152,378 are women of childbearing age, with an estimated 22,627 pregnancies. Healthcare services are provided by 1 teaching hospital, 1 primary hospital, 26 health centers, and 130 health posts. The data collection took place at health facilities in North and Sheko districts, as well as at the Mizan-Aman town administration.

### Populations

The source population comprised pregnant women attending Antenatal care (ANC) follow-ups at selected health facilities during the data collection period. The study population included pregnant women meeting the inclusion criteria during the same period. Cases were pregnant women diagnosed with HBV infection (positive HBsAg test) attending ANC at the selected health institution. Controls were pregnant women without HBV infection (negative HBsAg test) attending ANC at the same institution.

Inclusion Criteria

✓ Pregnant women attending antenatal care (ANC) follow-ups at selected health facilities during the data collection period.
✓ Pregnant women who underwent HBV testing and met the study criteria.

Exclusion Criteria

✓ Pregnant women who did not provide written informed consent.

### Sample size determination

The sample size was calculated using Open Epi version 3.01 software by considering the following assumptions. The expected proportion of multiple sexual partners among HBV-infected cases and controls was 68.9% and 47.5% respectively (18), 95% CI, 80% power, and a case-to-control ratio of 1:2. The sample size was 207. Thus, after considering the 10% non-response rate, the total study participants were 228 (76 cases and 152 controls).

### Sampling procedure

North and Sheko districts, along with the Mizan-Aman town administration, were chosen at random from a total of six districts and one town administration within the Bench Sheko zone. All health centers and hospitals providing ANC in these areas were included. The sample size was allocated to each institution based on average monthly client flow. Pregnant women's serologic status was checked from ANC registration books, categorizing them into case and control groups. For each case, the next two controls were consecutively interviewed until reaching the required sample size.

### Study variables

The dependent variable was HBV infection. The independent variables included socio-demographic factors (age, residence, education, marital status, and occupation), reproductive health history (unsafe abortion, pregnancies, delivery location and method, pregnancy complications), medical/surgical history (hospitalization, surgery, unsafe tooth extraction, blood transfusion, STI/HIV history), behavioral factors (multiple sexual partners, household HBV contact, sharing items), and cultural/cosmetic practices (tribal scarification, ear piercing, tattoo, traditional removal of the uvula/tonsil, and circumcision/genital mutilation) (Supplementary Table S1).

### Data collection tools and procedures

Data collection involved face-to-face interviews using a structured and pre-tested questionnaire adapted from relevant literature and tailored to the local context. The questionnaire, initially in English, was translated to Amharic and back to English for consistency. It covered socio-demographic, reproductive, medical, surgical, behavioral, cultural, and cosmetic aspects. Medical charts of the participants were reviewed to collect only the HBsAg status, as routine screening was conducted for all pregnant women attending antenatal care. A pretest was conducted on 5% of pregnant women (4 cases and 8 controls) at a health institution out of the actual study area. Training that lasted one day was provided to data collectors and supervisors about data collection procedures and quality assurance.

### Statistical analysis

Data coding and entry were performed using Epi-Data version 3.1 and then analyzed using SPSS version 21. Descriptive statistics, including frequencies and percentages, were performed, and findings were presented through tables. Each potential independent factor related to HBV infection underwent bivariate logistic regression analysis. To prevent overfitting, only variables showing a *p*-value <0.25 in the bivariate analysis were included in the multivariable logistic regression analysis. Those with a *p*-value <0.05 in the multivariable analysis were deemed determinants of HBV. The model's independent variables showed an acceptable variance inflation factor (VIF) indicating low multicollinearity (VIF <2), and the model fit well, as indicated by the Hosmer-Lemeshow goodness of fit test (*p* = 0.566).

## Results

### Socio-demographic characteristics

A total of 212 pregnant women participated in the study, including 71 cases and 141 controls, with response rates of 93.4% and 92.8%, respectively. The mean age was 27.6 ± 6.4 years, with cases slightly older (28.1 ± 6.9) than controls (27.3 ± 6.2). Among cases, 16 (22.5%) had no formal education, compared to 14 (9.9%) of controls. Sixty-one (85.9%) were married, while 107 (75.8%) of controls were married. Thirty-one (43.6%) of cases were housewives, compared to 73 (51.7%) of controls (Table 1).

**Table 1:** Socio-demographic characteristics of study participants in Bench Sheko zone, Southwest Ethiopia.

Variables	Categories	Cases *n* (%)	Controls *n* (%)	Total *n* (%)	*P-value*
Age (mean ± SD) years		28.1 ± 6.9	27.3 ± 6.2	27.6 ± 6.4	0.232
Education	No formal education	16 (22.5)	14 (9.9)	30 (14.2)	0.023
	Primary school	31 (43.6)	60 (42.5)	91 (42.9)	
	Secondary school	17 (23.9)	35 (24.8)	52 (24.5)	
	Diploma and above	7 (9.8)	32 (22.6)	39 (18.4)	
Marital status	Unmarried	10 (14.1)	34 (24.1)	44 (20.8)	0.178
	Married	61 (85.9)	107 (75.9)	168 (79.2)	
Occupation	Housewife	31 (43.7)	73 (51.7)	104 (49.1)	0.108
	Self-employee	14 (19.7)	23 (16.3)	37 (17.5)	
	Private employee	14 (19.7)	16 (11.3)	30 (21.3)	
	Government employee	11 (15.5)	17 (12)	28 (13.2)	
	Others	1 (1.4)	12 (8.5)	13 (6.1)	
Residence	Urban	40 (56.3)	59 (41.8)	99 (46.7)	0.032
	Rural	31 (43.7)	82 (58.2)	113 (53.3)	

### Reproductive health history

Among cases, 38 (53.5%) were experiencing their first pregnancy, compared to 48 (34%) of controls. Thirteen (39.3%) of cases and 27 (23.6%) of controls gave their recent birth at home. Spontaneous vaginal delivery was more common among controls (81, 87%) than cases (23, 69.6%). Pregnancy-related complications affected 15 (21.2%) of cases and 25 (17.7%) of controls. Additionally, 15 (10.7%) of controls and 16 (22.5%) of cases had a history of unsafe abortion. Controls were more likely to have heard about HBV infection (102, 72.3%) compared to cases (41, 57.7%) (Table 2).

**Table 2:** Reproductive health-related factors of study participants in Bench Sheko zone, Southwest Ethiopia.

Variables	Categories	Cases *n* (%)	Controls *n* (%)	Total *n* (%)	*P-value*
Number of pregnancies	Primigravida	38 (53.5)	48 (34)	86 (40.6)	0.007
	Multigravida	33 (46.5)	93 (66)	126 (59.4)	
Previous place of delivery	Home	13 (39.4)	27 (23.6)	40 (31.7)	0.188
	Health institution	20 (60.6)	66 (76.4)	86 (68.3)	
Mode of delivery	Vaginal	23 (69.6)	81 (87.1)	104 (82.5)	0.563
	Cesarean section	10 (30.4)	12 (12.9)	22 (17.5)	
Pregnancy-related complications	Yes	15 (21.2)	25 (17.7)	40 (18.8)	0.337
	No	56 (78.8)	116 (82.3)	172 (81.2)	
History of unsafe abortion	Yes	16 (22.5)	15 (10.7)	31 (14.6)	0.025
	No	55 (77.5)	126 (89.3)	181 (85.4)	
Number of unsafe abortions	Once	11 (68.8)	9 (60)	20 (64.5)	0.479
	More than once	5 (31.2)	6 (40)	11 (35.5)	
Ever heard about HBV	Yes	41 (57.7)	102 (72.3)	143 (67.5)	0.024
	No	30 (42.2)	39 (27.6)	69 (32.5)	
HBV vaccination	Yes	7 (9.9)	11 (7.8)	18 (8.5)	0.798
	No	64 (90.1)	130 (92.2)	194 (91.5)	

### Behavioral and cultural-related characteristics

Participants with a history of sharing sharp materials were higher among cases (51, 71.8%) compared to controls (26, 18.4%). Cases had a higher proportion of history of contact with HBV-infected persons (12, 16.9%) compared to controls (11, 7.8%). Cases had a higher proportion of tribal scarification (25, 35.2%) compared to controls (25, 17.7%). Similarly, cases had a lower proportion of removal of the uvula (3, 4.2%) compared to controls (24, 17%) (Table 3).

**Table 3:** Behavioral and cultural-related factors of study participants in Bench Sheko zone, Southwest Ethiopia.

Variables	Categories	Cases *n* (%)	Controls *n* (%)	Total *n* (%)	*P*-value
Sharing sharp object/materials	Yes	51 (71.8)	26 (18.4)	77 (36.4)	<0.001
	No	20 (28.2)	115 (81.6)	135 (63.6)	
Sharing toothbrush	Yes	3 (4.2)	1 (0.7)	4 (1.9)	0.342
	No	68 (95.7)	140 (99.2)	208 (98.1)	
History of multiple sexual partners	Yes	14 (19.7)	17 (12)	31 (14.7)	0.101
	No	57 (80.2)	124 (87.9)	181 (85.3)	
History of contact with HBV infected person	Yes	12 (16.9)	11 (7.8)	23 (10.9)	0.001
	No	59 (83.1)	130 (92.2)	189 (89.1)	
Have tattoo	Yes	9 (12.7)	19 (13.4)	28 (13.2)	0.528
	No	62 (73.3)	122 (86.5)	184 (86.8)	
Have tribal scar or scarification	Yes	25 (35.2)	25 (17.7)	50 (23.6)	0.004
	No	46 (64.7)	116 (82.2)	162 (76.4)	
Have ear piercing	Yes	63 (88.7)	132 (93.6)	195 (92)	0.168
	No	8 (11.3)	9 (6.4)	17 (8)	
History of removal of the uvula	Yes	3 (4.2)	24 (17)	27 (13.3)	0.235
	No	68 (95.7)	116 (82.2)	184 (86.7)	
History of removal of the tonsil	Yes	12 (16.9)	27 (19.2)	39 (18.4)	0.422
	No	59 (83.1)	114 (80.8)	173 (81.6)	
History of circumcision or genital mutilation	Yes	5 (7.1)	16 (11.3)	21 (9.9)	0.231
	No	66 (92.9)	125 (86.7)	191 (90.1)	

### Medical and surgical history

Cases had a higher history of surgery (12, 16.9%) compared to controls (15, 10.6%). Unsafe tooth extraction was more common among cases (16, 22.5%) than controls (8, 5.6%). Sexually transmitted diseases were reported more frequently among cases (15, 21%) compared to controls (15, 10.6%). Additionally, the HIV/AIDS positivity rate was higher among cases (8, 11.2%) than controls (6, 4.3%) (Table 4).

**Table 4:** Medical and surgical history of study participants in bench sheko zone, southwest Ethiopia.

Variables	Categories	Cases *n* (%)	Controls *n* (%)	Total *n* (%)	*P-value*
History of any surgery	Yes	12 (16.9)	15 (10.6)	27 (12.8)	0.142
	No	59 (83.1)	126 (89.3)	185 (87.2)	
History of unsafe tooth extraction	Yes	16 (22.5)	8 (5.6)	24 (11.4)	<0.001
	No	55 (77.4)	133 (94.3)	188 (88.6)	
History of hospital admission	Yes	12 (16.9)	29 (20.6)	41 (19.4)	0.329
	No	59 (83.1)	112 (89.4)	171 (80.6)	
History of blood transfusion	Yes	3 (4.2)	12 (8.5)	15 (7.1)	0.196
	No	68 (95.8)	129 (91.5)	197 (92.9)	
History of STD	No	15 (21.1)	15 (10.6)	30 (14.2)	0.034
	Yes	56 (78.9)	126 (89.4)	182 (85.8)	
Current HIV status	Positive	8 (11.3)	6 (4.3)	14 (6.6)	0.043
	Negative	63 (88.7)	135 (95.7)	198 (93.4)	

### Determinants of HBV infection

After adjusting for confounding factors, the study identified several significant determinants of HBV infection: pregnant women without formal education had 4.9 times higher odds of HBV infection compared to those with diplomas or higher education (AOR = 4.94, 95% CI: 2.01, 8.29; *P = 0.007*). Urban residents were 2.6 times more likely to have HBV than rural residents (AOR = 2.56, 95% CI: 1.43, 6.86; *P = 0.010*). A history of unsafe abortion increased the odds of HBV infection by 3.9 times (AOR = 3.87, 95% CI: 2.17, 6.98; *P < 0.001*). Sharing sharp materials like syringes and needles increased the odds by 8.4 times (AOR = 8.43, 95% CI: 5.54, 10.9; *P < 0.001*). Contact with HBV-infected persons in the family raised the odds by 2.2 times (AOR = 2.18, 95% CI: 1.72, 4.87; *P < 0.001*). Tribal scarification increased the odds by 3.2 times (AOR = 3.23, 95% CI: 1.24, 8.91; *P = 0.017*). The history of unsafe tooth extraction increased the odds by 4.5 times (AOR = 4.52, 95% CI: 2.18, 9.76; *P = 0.039*) (Table 5).

**Table 5:** Determinants of HBV infection among study participants in Bench Sheko zone, Southwest Ethiopia.

Variables	Categories	COR (95% CI)	AOR (95% CI)	*P-value*
Education	No formal education	5.22 (1.76, 15.5)**	4.94 (2.01, 8.29)	0.007
	Primary school	2.36 (0.94, 5.96)	3.22 (0.71, 5.68)	0.127
	Secondary school	2.22 (0.81, 6.04)	3.05 (0.82, 7.52)	0.135
	Diploma and above	1	1	
Residence	Urban	1.79 (1.01, 3.19)*	2.56 (1.43, 6.86)	0.010
	Rural	1	1	
Number of pregnancies	Primigravida	2.23 (1.54, 6.77)	3.34 (0.99, 7.89)	0.342
	Multigravida	1	1	
History of unsafe abortion	Yes	2.44 (1.13, 5.29)**	3.87 (2.17, 6.98)	<0.001
	No	1	1	
Heard about HBV	Yes	0.52 (0.28, 0.95)*	0.38 (0.10, 1.24)	0.059
	No	1	1	
Sharing sharp materials	Yes	11.3 (5.75, 17.4)**	8.43 (5.54, 10.9)	<0.001
	No	1	1	
Contact with HBV infected person	Yes	2.40 (1.15, 5.46)**	2.18 (1.72, 4.87)	<0.001
	No	1	1	
Having tribal scar	Yes	2.52 (1.31, 4.84)*	3.23 (1.24, 8.91)	0.017
	No	1	1	
History of unsafe tooth extraction	Yes	4.84 (1.95, 11.9)*	4.52 (2.18, 9.76)	0.039
	No	1	1	
History of sexually transmitted disease	Yes	2.25 (1.02, 4.94)	1.48 (0.97, 4.42)	0.260
	No	1	1	
Current HIV status	Positive	2.86 (0.95, 8.58)	2.08 (0.33, 12.9)	0.432
	Negative	1	1	

1.00, reference value.

* *p*-value <0.05.

** *p*-value <0.001; COR, crude odds ratio; AOR, adjusted odds ratio; CI, confidence interval.

## Discussion

In Ethiopia, despite screening efforts, the health system lacks focus on identifying the root causes of HBV infection (6, 17). The study aimed to identify potential determinants of HBV infection among pregnant women. The study identified key risk factors for HBV infection, such as education level, residence, unsafe abortion history, sharing sharp materials, contact with hepatitis patients, tribal scars, and unsafe tooth extraction. Understanding these factors helps target preventive measures and interventions more effectively.

In line with the findings of previous studies, there was a positive association between level of education and HBV infection (14, 19). Low literacy levels may contribute to HBV transmission due to limited health literacy, barriers to healthcare access, socioeconomic challenges, risky behaviors, and stigma. Enhancing education, improving healthcare access, and addressing social determinants are crucial for reducing HBV infection among illiterate populations.

Urban participants exhibited higher risk of HBV infection compared to rural participants, consistent with previous studies (20, 21). This phenomenon may be due to high urban population density, which increases transmission through close contact and shared resources, along with significant population mobility that spreads HBV among diverse groups. Additionally, higher rates of risky behaviors like unsafe injections and sharing sharp materials, coupled with socioeconomic disparities affecting access to health resources, contribute to the increased risk (21).

A history of unsafe abortion was significantly correlated with HBV infection, which aligns with studies in Ethiopia (13, 22, 23) and Nigeria (18). The increased risk of HBV transmission during abortions outside healthcare facilities is due to inadequate infection prevention measures, including the use of contaminated instruments, and the absence of medical supervision and sterile conditions, heightening the likelihood of exposure.

Participants who shared sharp materials had a greater chance of having HBV infection. This finding is consistent with other studies (15, 24). Sharing sharp materials, such as needles or razors, significantly increases the risk of contracting HBV due to direct blood-to-blood contact. This close exchange of bodily fluids provides an efficient pathway for the transmission of the virus, elevating the likelihood of infection.

Participants with a history of family contact with hepatitis patients exhibited higher odds of HBV infection compared to those without such contact, aligning with findings from studies conducted elsewhere (12, 13, 15, 18, 25, 26). The increased risk of HBV infection may be due to unprotected caregiving contact and limited awareness of transmission modes and prevention measures, leading to behaviors that facilitate virus transmission, such as sharing personal items or engaging in unprotected sexual activity.

The history of tribal scarification is associated with HBV infection in this study. This finding is supported by studies conducted elsewhere (27, 28). The increased likelihood of risk can be elucidated by the utilization of inadequately sterilized equipment throughout the procedure (28). This increased risk arises from the potential for pathogens and contaminants to persist on improperly cleaned instruments, thus increasing the probability of infections or complications for individuals undergoing the procedure.

Pregnant women with a history of unsafe tooth extraction exhibited 4.5 times higher odds of HBV infection compared to those without such a history, consistent with studies in Ethiopia (12, 29), and Saudi Arabia (30). The increased risk may arise from inadequate infection control measures during unsafe tooth extraction procedures, posing threats to both healthcare workers and patients. Additionally, traditional tooth removal methods that involve non-sterile materials could further contribute to HBV transmission.

### Limitations of the study

Blood samples weren't directly collected from participants due to financial limitations, so HBV status relied on secondary data. Using only the HBsAg marker to define the clinical status of pregnant women is a limitation. Including markers like anti-HBe antibodies and HBeAg could have provided a more detailed assessment of HBsAg-positive women. Exposure variables, like STI history, were symptom-based, possibly leading to recall bias. Sensitive questions, such as those about multiple partners, might have faced social desirability bias.

## Conclusion

The study identifies several determinants of HBV infection in pregnant women, including no formal education, urban residency, history of unsafe abortion, sharing sharp materials, contact with HBV-infected family members, tribal scarification, and history of unsafe tooth extraction. Effective reduction of HBV risk relies on targeted interventions, such as health education to increase awareness, ensuring safe healthcare practices, promoting HBV vaccination, and offering support to high-risk pregnant women. Addressing these factors can significantly lower HBV infection rates among pregnant women and improve maternal and child health outcomes.

## Data Availability

The raw data supporting the conclusions of this article will be made available by the authors, without undue reservation.
