# Supplementary material for: Determinants of hepatitis B virus infection among pregnant women in Bench Sheko zone, Southwest Ethiopia: a case-control study
Source: Front Glob Womens Health. 2024 Oct 14;5:1453231. doi: 10.3389/fgwh.2024.1453231 (PMC11513388; doi:10.3389/fgwh.2024.1453231)
Supplement: Supplementary file 1 [file Table1.docx]

Supplementary table 1: Operational definitions of the study variables

| Variables | Definitions |
| --- | --- |
| Gravidity | Primigravida is a woman pregnant for the first time, while multigravida refers to a woman who has been pregnant more than once, regardless of pregnancy outcomes. |
| Mode of delivery | Vaginal delivery is when the baby is born through the birth canal, while a C-section involves surgically delivering the baby through incisions in the abdomen and uterus. |
| Pregnancy related complications | Common complications affecting maternal, or baby health include gestational diabetes, preeclampsia, preterm labor, miscarriage, placenta previa, ectopic pregnancy, and anemia. |
| Unsafe abortion | Unsafe abortion is a pregnancy termination before 28 weeks of gestation performed by unskilled individuals or in substandard medical environments. |
| Sharing sharp object/materials | Sharing sharp objects or materials, such as needles or blades, poses significant health risks, including the transmission of infections (e.g., HIV, hepatitis). |
| Multiple sexual partners | Multiple sexual partners refer to engaging in sexual relationships with more than one person simultaneously or over a given period. |
| Tribal scarification | Tribal scarification is the intentional creation of scars on the skin for cultural, spiritual, or aesthetic reasons, often signifying identity, status, or rites of passage within a community. |
| Circumcision or genital mutilation | Circumcision is the removal of the foreskin from the penis, while genital mutilation involves altering or injuring the genital organs for non-medical reasons, often resulting in harm or complications. |
| Unsafe tooth extraction | Unsafe tooth extraction is the removal of a tooth in unhygienic conditions or by an untrained person, leading to infections and other health risks. |
